# Complex Phase-Fluctuation Effects Correlated with Granularity in Superconducting NbN Nanofilms

**DOI:** 10.3390/nano12234109

**Published:** 2022-11-22

**Authors:** Meenakshi Sharma, Manju Singh, Rajib K. Rakshit, Surinder P. Singh, Matteo Fretto, Natascia De Leo, Andrea Perali, Nicola Pinto

**Affiliations:** 1 School of Science and Technology, University of Camerino, 62032 Camerino, Italy; 2 CNR-Institute for the Study of Nanostructured Materials, 40129 Bologna, Italy; 3 National Physical Laboratory, CSIR, New Delhi 110012, India; 4 Advanced Materials Metrology and Life Science Division, INRiM, 10135 Torino, Italy; 5 School of Pharmacy, Physics Unit, University of Camerino, 62032 Camerino, Italy

**Keywords:** NbN, ultrathin films, BKT transition, phase slips, granular superconductivity

## Abstract

Superconducting nanofilms are tunable systems that can host a 3D–2D dimensional crossover leading to the Berezinskii–Kosterlitz–Thouless (BKT) superconducting transition approaching the 2D regime. Reducing the dimensionality further, from 2D to quasi-1D superconducting nanostructures with disorder, can generate quantum and thermal phase slips (PS) of the order parameter. Both BKT and PS are complex phase-fluctuation phenomena of difficult experiments. We characterized superconducting NbN nanofilms thinner than 15 nm, on different substrates, by temperature-dependent resistivity and current–voltage (I-V) characteristics. Our measurements evidence clear features related to the emergence of BKT transition and PS events. The contemporary observation in the same system of BKT transition and PS events, and their tunable evolution in temperature and thickness was explained as due to the nano-conducting paths forming in a granular NbN system. In one of the investigated samples, we were able to trace and characterize the continuous evolution in temperature from quantum to thermal PS. Our analysis established that the detected complex phase phenomena are strongly related to the interplay between the typical size of the nano-conductive paths and the superconducting coherence length.

## 1. Introduction

Effects related to thermal and quantum fluctuations in low-dimensional superconductors, such as phase slips [1,2,3,4,5], quantum criticality [6], superconductor-insulator transition [7] and quantum-phase transitions [8,9], have been studied for several decades. These quantum and many-body effects are controlled by several film properties, such as spatial dimensions, electronic disorder and structural inhomogeneities [10,11].

In recent years, the scientific interest has been focused on quasi 2D and 1D systems, where the presence of resistive states close to the superconducting transition temperature $T_{c}$ has been found to produce detectable effects in the transport properties [10,11].

In such systems, the emerging resistive states are a fundamental phenomenon, which involves the understanding of advanced concepts as topological excitations, phase disorder and interplay between different length scales of the superconducting state. These have important applications in the field of quantum technologies, ultrasonic detectors of radiation, single photon detector and nanocalorimeters [10,12].

Efforts have been made to study low-dimensional systems theoretically, and from the experimental side, the attention has been focused on thin films in which a crossover from 3D to 2D Berezinskii–Kosterlitz–Thouless (BKT) transition [13,14,15,16] occurs, lowering the thickness to few nanometers, as is the case, for instance, for NbN films [15,17,18,19,20].

A superconducting-to-normal-state transition in a 2D-XY model was introduced to explain the formation of thermally excited vortex-anti-vortex pairs (VAP) in ultrathin films, even in the absence of an applied magnetic field [13], resulting in the BKT topological phase transition [13,21]. The nature of a BKT transition is completely different from the standard second-order phase transition given by the Landau paradigm. It is driven by the binding of topological excitations without any symmetry breaking associated with the onset of the order parameter [22].

A BKT-like transition is expected to occur even in dirty superconductors, at a reduced film thickness *d*, under some physical constraints. A required condition is that the Pearl length $\Lambda =2\lambda^{2}/d$ (λ being the London penetration depth), exceeds the sample size with negligible screening effects due to charged supercurrents [23]. Furthermore, λ≫w or $d\ll \xi_{GL}$ ($\xi_{GL}$ being the Gintzburg–Landau coherence length) must be fulfilled in order to detect BKT effects, though some experiments have reported the BKT transition is also outside the theoretically established limits [24].

To observe this remarkable phenomenon in real systems, two approaches have been explored. In the first, the BKT theory predicts a universal jump in the film’s superfluid stiffness, $n_{s}$, at the characteristic temperature $T_{BKT}<T_{MF}$, $T_{MF}$ being the mean-field superconducting transition temperature. This jump is related to the VAP binding through a logarithmic interaction potential between free vortices. Sourced current can break bound pairs, producing free vortices, inducing nonlinear effects in I-V curves [24]. For the second approach, transition is observed in the correlation length, which diverges exponentially at $T_{BKT}$, in contrast to the power-law dependence expected within the Ginzburg–Landau (GL) theory [25,26].

When further reducing film size, fluctuations can result in the formation of multiple resistive states in I-V curves, at both low and high *T* ranges. These intermediate states form due to the change in phase of the order parameter by 2π, and they will result in a discontinuous voltage jump, forming phase slips. These PS are characteristic of a quasi-1D system, which form by a river of fast moving vortices (kinematic vortices) driven by the topological excitations, annihilating in the middle of the sample [13,27]. However, several superconductors have been explored experimentally to study such topological effects, including NbSe${}_{2}$ [27], NbN [13], Nb${}_{2}$N [11], Nb [28] and many more.

In particular, NbN is a known and well-studied material, belonging to the family of strongly coupled type-II superconductors, and it is potentially interesting for several technological applications, due to its relatively high value of bulk $T_{c}$ (≃16 K). Its small coherence length, ξ (≈4 nm), requires fabrication of extremely thin films (few nanometers of thickness) with fine control of their properties to achieve the 2D superconducting regime [29].

In this work, a detailed experimental study was carried out about the electrical properties of superconducting, NbN ultra-thin films, aimed at investigating the crossover regime from a quasi-2D BKT phenomenon to a quasi-1D PS mechanism. Outcomes evidenced the presence of large fluctuation effects mostly close to $T_{c}$, and an exponential decrease at lower temperatures. In one case, the freezing of thermal fluctuations at the lowest temperatures gave rise to a quantum phase slip (QPS) phenomenon, whereas in the other case two distinct resistive transitions, below $T_{c}$, were detected for one of the thinnest NbN films, suggesting the unexpected coexistence of a BKT transition and phase-slip phenomena at the dimensional crossover from 2D to 1D.

Therefore, findings on superconducting to resistive-state transition features in investigated thin films of NbN have been questioning if their origin is due to thermal fluctuations (e.g., thermal PS), quantum fluctuations (e.g., QPS) [30] or a proximity effect mechanism among coupled nano-sized superconducting grains [11]. Our study suggests that the thickness threshold where quantum phase fluctuation effects can start to appear is not yet clearly defined, since BKT transition or QPS phenomena have been detected even in NbN films that are nominally 10 nm thick. Hence, specific conditions to detect BKT transitions and PS events in quasi-2D systems, especially those being granular in nature, deserve to be further studied both theoretically and experimentally.

## 2. Materials And Methods

### 2.1. Deposition

NbN films with nominal thicknesses of 5, 10 and 15 nm were deposited on several substrates, such as MgO, Al${}_{2}$O${}_{3}$ and SiO${}_{2}$ (see Table 1), by using DC magnetron sputtering. The optimized deposition rate was ≃0.4 nm/s, at a substrate temperature of 600 °C and at 200 W of discharge power. The N${}_{2}$/Ar ratio was fixed at 1:7 during the deposition process.

### 2.2. Fabrication of the Hall Bar

**Figure 1:**
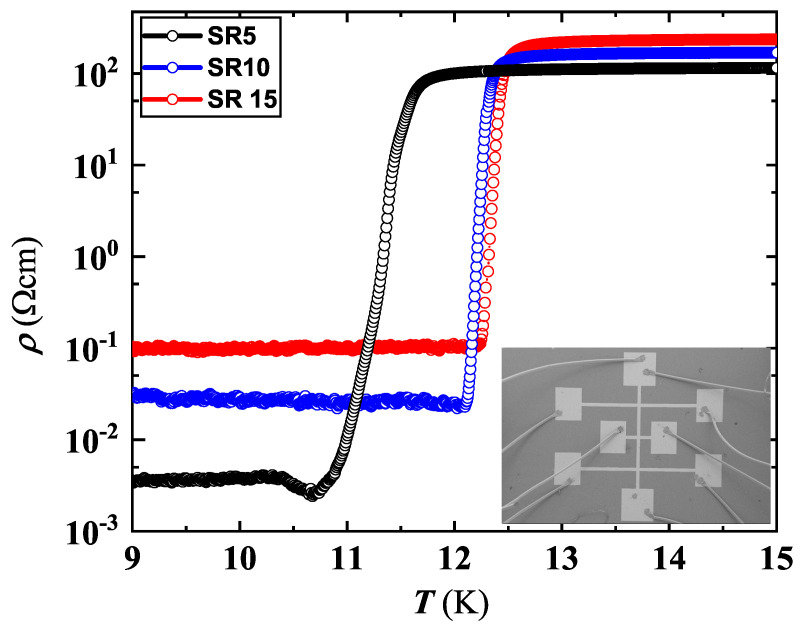
NbN resistivity behavior around $T_{c}$, for the set deposited on the Al${}_{2}$O${}_{3}$ r−cut substrate (see Table 1). Inset: Scanning electron microscopy of a typical Hall bar shaped film with Al wires bonded to sample pads. Current carrying contacts were located on the top and bottom, along the vertical line; and a couple of lateral contacts from the same side were used to detect the voltage drop.

For the electrical characterization, a suitable 8-contact Hall bar geometry was designed (see inset in Figure 1), by using LibreCAD software. The corresponding optical mask was realized by using direct laser lithography (at a wavelength of 375 nm) exploiting a µPG101 laser writer from Heidelberg Instruments. The Hall bar length was fixed at 1000 µm, and bar widths of 10 and 50 µm were chosen. The Hall bars were patterned by optical lithography with a KarlSuss mask aligner (mod. MJB3) by using a reversible photoresist (AZ 5214E Photoresists MicroChemicals GmbH), spun at 4000 rpm, resulting in a nominal thickness of 1.2 μm. Later, an etching step was exploited to define the final geometry of the NbN Hall bars. In particular, the etching process was carried out by using deep reactive ion etching (PlasmaPro 100 Cobra Inductively Coupled Plasma Etching System, Oxford Instrument). The fabrication parameters (etching time, ICP power, substrate temperature, etc.) were optimized while taking into account the film thicknesses and typology of substrates. The best etching selectivity between the optical resist and the NbN thin film was obtained with a mixture of CF4 and Ar with fluxes of 90 and 10 sccm, respectively, at an operating pressure of 50 mTorr. ICP power and RIE RF power were 500 and 30 W, respectively. All the samples were cleaned with a light-oxygen plasma before the etching, to eliminate any organic or lithographic residuals. Further, the samples were glued to a copper holder with a high-thermal-conductivity glue (GE Varnish) in order to have better temperature control and stability in the cryostat. Aluminum wires (see inset of Figure 1) were bridge bonded to the pads of the Hall bar and to the pads of a small printed circuit board, placed close to the sample. A scanning electron microscope (SEM) was used for the visual inspection to check the intermediate steps of the fabrication process.

### 2.3. Electrical Characterization

Resistivity, ρ(T), and current–voltage (I-V) characteristics were measured as a function of temperature in a liquid-free He cryostat (Advanced Research System mod. DE210) equipped with two Si diode thermometers (Lakeshore mod. DT-670)—one out of two calibrated [31,32]. A temperature controller Lakeshore mod. 332 was used to read the temperature of the uncalibrated thermometer, thermally anchored to the second stage of the cryostat. The film temperature was measured by using one channel of a double source-meter (Keysight mod. B2912A). The other channel of the instrument was earmarked for electrical characterization of NbN film properties (ρ and I-V) in the 4-contact geometry, sourcing the current (0.1 ÷ 100 μA, typically 1 μA for ρ) and detecting the voltage drop. Either dc or a pulsed mode technique was used.

I-V characteristics was carried out both in dc mode [33] and in pulse mode. For measurements executed by the pulse-mode technique [34], a sweep of current pulses of increasing intensities, ranging from tens of μA to few mA, each of duration 1.1 ms, was used. Due to the high thermal inertia of the cryostat, data were collected without any thermal stabilization in the whole range of temperatures (≈5÷300 K) upon sample cooling. The maximum *T* change, detected at the lowest *T* during data acquisition (e.g., ρ), was around 15 mK. For each data point of the resistivity curve, typically 30 values were averaged, and a suitably selection of the working parameters of the source-meter allowed us to capture several values during the transition from the superconducting to the normal state. For I-V characteristics, typically 200 points were collected in a few seconds for each curve, and the maximum *T* variation during each I-V curve acquisition was ≲50 mK.

## 3. Results

### 3.1. Superconducting State Properties

NbN film properties were investigated by resistivity and current–voltage characteristics as a function of the temperature.

At the superconducting (SC) transition, the investigated films showed a resistivity jump spanning from ≈2 to ≈5 orders of magnitude; $T_{c}$ depends on the film thickness and substrate type, and its value rapidly decreases at d≤10 nm (see Table 1).

The ρ(T) curves around $T_{c}$ for the set deposited on the Al${}_{2}$O${}_{3}$ r-cut substrate is reproduced in Figure 1. The SC transition lowers with the decrease of the film thickness, becoming substantial when passing from d=10 to d=5 nm. The normal state resistivity does not show, in general, a clear correlation with the value of *d*, appearing even reversed with respect to the *d* value, for the set deposited on the r-cut sapphire (Figure 1). However, normal-state resistivity variation is confined to within a half order of magnitude for 5≤d≤15 nm. The behavior of ρ(T) for SR5 is analyzed in detail in the next section.

**Figure 2:**
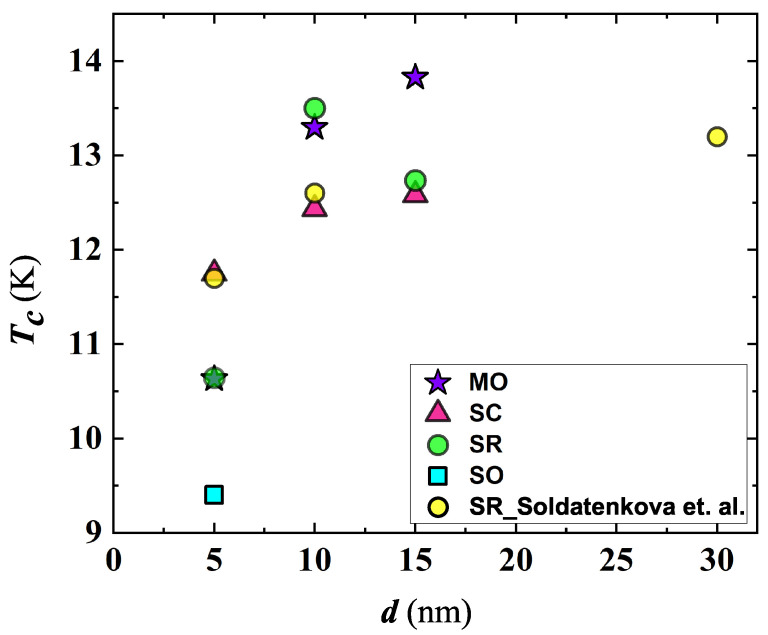
Thickness dependence of the superconducting transition temperature of NbN films deposited on MgO (MO), SiO${}_{2}$ (SO) and Al${}_{2}$O${}_{3}$ substrates, both c-cut (SC) and r-cut (SR) types. For comparison, $T_{c}$ data of films deposited on Al${}_{2}$O${}_{3}$ r-cut substrates by Soldatenkova et al. [37] were plotted.

Generally, a higher $T_{c}$ together with a narrow SC transition width, $\Delta T_{c}$, were measured on MgO substrates above d=10 nm (Figure 2 and Table 1), whereas a consistent worsening of these parameters occurred on SiO${}_{2}$ (this behavior was detected also in films of higher thicknesses not reported in the present work). Intermediate values of both $T_{c}$ and $\Delta T_{c}$ were measured on Al${}_{2}$O${}_{3}$ substrates; $T_{c}$ on the c-cut type was slightly higher at d>5 nm (Figure 2). Compared to NbN films deposited on Al${}_{2}$O${}_{3}$ r-cut, those on the c-cut type showed a wider range of $T_{c}$ variation with *d* and a tendency toward the narrowing of $\Delta T_{c}$ (Table 1). An increase in *d* denotes an improvement in the film’s quality, in agreement with findings reported by other groups [35,36].

While the general trend of $T_{c}$ as a function of *d* was reported in the literature by several groups [38,39], $T_{c}$ spreading depends also on the crystal structure [40], deposition technique, the partial pressure of nitrogen used during the film fabrication process, etc., making a direct comparison of results measured by different groups difficult [14,38,39,41]. Anyway, taking into account NbN films deposited on Al${}_{2}$O${}_{3}$ r-cut substrates, our $T_{c}$ values, while appearing scattered at d≤10 nm, follow a trend similar to that found by Soldatenkova et al. on the same substrate type (see Figure 2) [37]. Scattering of $T_{c}$ in NbN films reflects inhomogeneity issues that are characteristic of this superconducting system [16].

**Figure 3:**
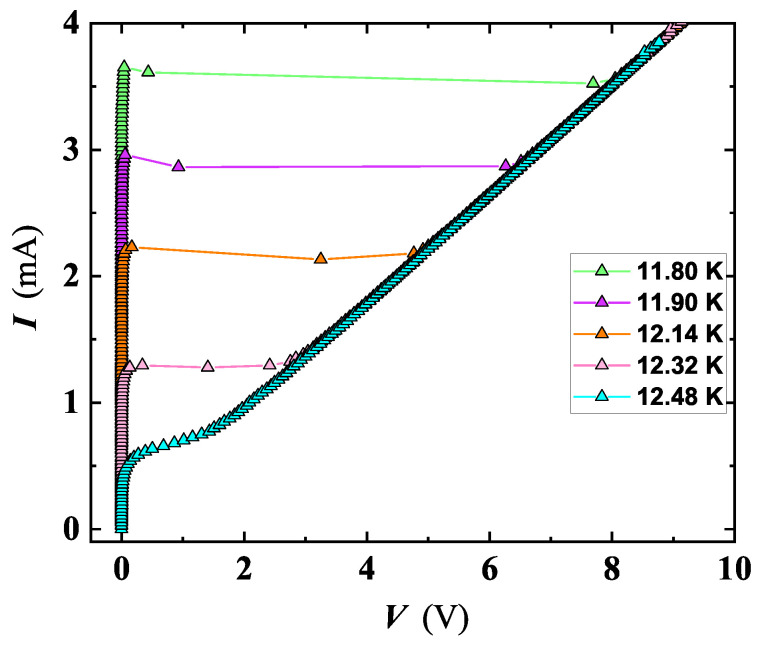
I-V curves of the film SC15, measured at several *T* close to the SC transition of $T_{c}$ = 12.73 K. Approaching $T_{c}$, the amplitude of the hysteresis between the sweep-up and sweep-down of the current narrows.

**Figure 4:**
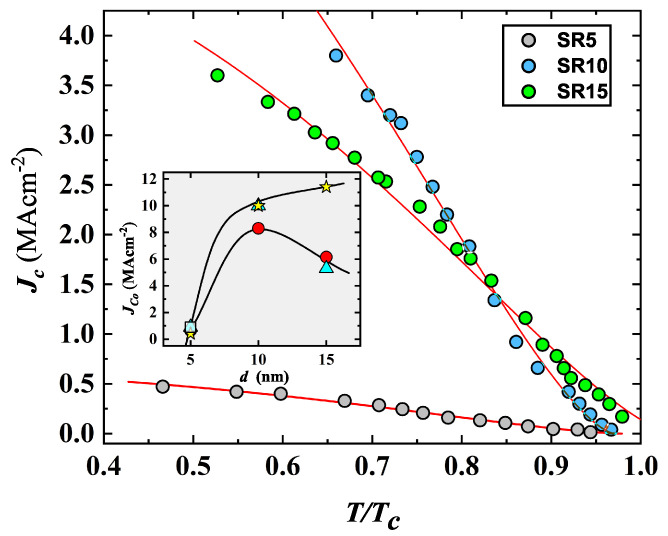
Current density as a function of the temperature, normalized to $T_{c}$, for the whole set of NbN films deposited on the Al${}_{2}$O${}_{3}$ r-cut substrate. Lines are the result of the least-squares fitting by the Ginzburg–Landau equation (see ref. [31]). Inset: thickness dependence of the critical current density at zero temperature, $J_{c0}$, for NbN films deposited on Al${}_{2}$O${}_{3}$ r-cut (circles), Al${}_{2}$O${}_{3}$ c-cut (triangles), MgO (stars), SiO${}_{2}$ (square).

The NbN film’s properties were studied further through current–voltage characteristics. Temperature dependent I-V curves exhibit hysteresis and a well-defined transition from the superconducting to the normal state for d>10 nm (Figure 3); and at 5 and 10 nm of thickness, several NbN films evidenced the presence of small steps along the SC transition branch of the I-V curve, which is better detailed in the next section. For these films, we assumed as critical current $I_{c}$, the value at which the first step occurs. The value of the critical current, $I_{c}$, progressively reduces with the rise in *T*, and smoothing of the transition occurs approaching $T_{c}$. The temperature dependence of the superconducting critical-current density, $J_{c}\left(T\right)$, for the set deposited on Al${}_{2}$O${}_{3}$ r-cut substrate (see Figure 4), was derived following the criterion and procedure reported in reference [31]. The $J_{c}$ values at 0 K (i.e., $J_{c0}$) were extracted by a least-squares fit using the Ginzburg–Landau equation [31].

The thickness dependence of $J_{c0}$ appears related to the substrate types exhibiting a bell shaped behavior on both types of sapphire substrates, and it continues to rise with film thickness on MgO (inset of Figure 4 and Table 1). It is worthwhile noting the drop in $J_{c0}$ at d=5 nm, up to about one order of magnitude, becoming practically independent on the substrate type, and little differences in $J_{c0}$ start to appear from d=10 nm (inset of Figure 4). The $J_{c0}$ values found in our films are similar to those found in thin films of NbN [42], and the bell shaped behavior was detected also in Nb [31].

### 3.2. Berezinskii–Kosterlitz–Thouless Transition

Experimental observation of a BKT transition in 2D systems is generally challenging due to the constraints on the film size in relation to the characteristic lengths of the superconductor. Concerning the Pearl length, the condition Λ>w must be fulfilled, *w* being a film dimension. Hence, assuming for NbN films a value of 0.5≲λ≲0.4μm for thicknesses 5≤d≤15 nm [43], we get values of Λ≈ 100 μm, Λ≈ 40 μm and Λ≈ 20 μm for d=5 nm, 10 nm and 15 nm. These values must be compared to the maximum physical dimension of the system, which for our films coincides with the width of the Hall bar (w=50 μm): for the Hall bar width of 10 μm, the condition is satisfied at any of the thicknesses here taken into account. Hence, while the 10 nm thick sample can be considered borderline, the 15 nm thick one appears out of range. Regarding the condition d≪ξ, it is worthwhile noting that being ξ≈ 4 nm [41], only NbN nanofilms, 5 nm thick, appear to be good candidates to exhibit a well-defined BKT transition.

Experimentally, we have investigated the signature of a BKT superconducting transition in ρ(T) curves and/or in I-V characteristics measured at several fixed *T*s [13,14,15,16].

**Figure 5:**
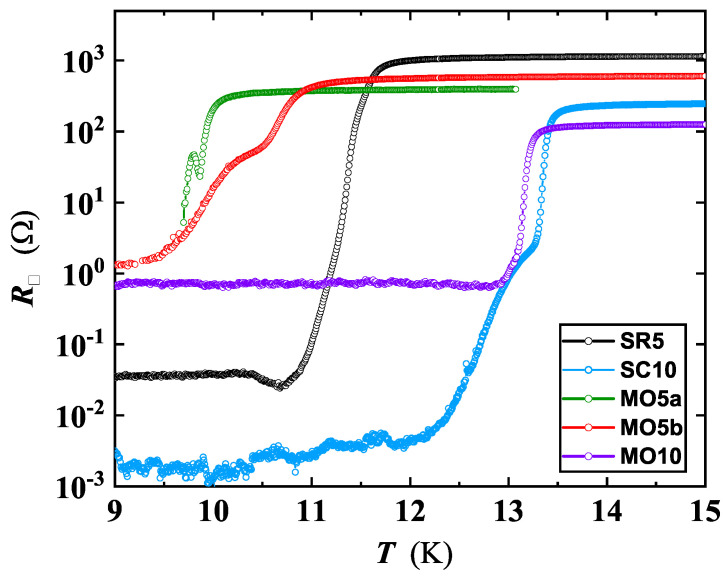
Temperature-dependent sheet resistance of ultrathin NbN films (5 and 10 nm of thickness), deposited on different substrate types, exhibiting quantum effects in 2D. The differences in the curve behavior of MO5a and MO5b (belonging to the same deposition run) are related to specific choices of process parameters during the Hall bar fabrication.

**Figure 6:**
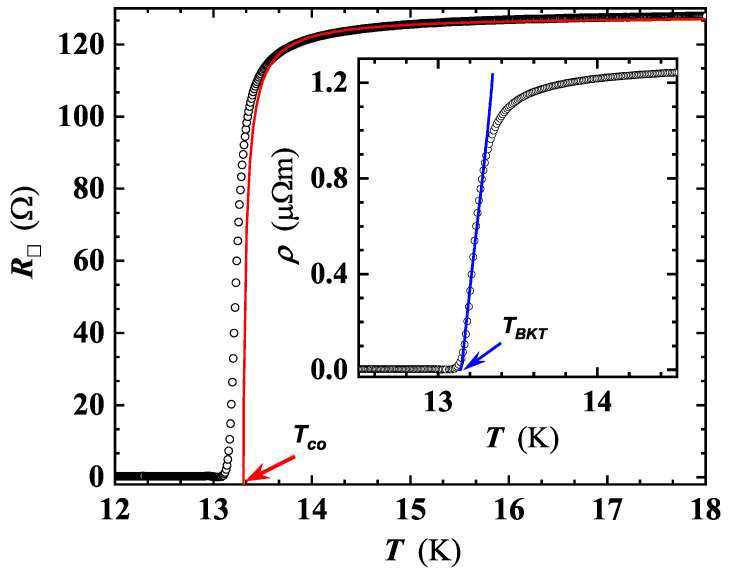
Temperature-dependent sheet resistance around the superconducting transition temperature for MO10 (see Table 2). The red line is the least-squares fit by using Equation (1). The value of $T_{c0}=13.31$ K (red arrow) is the intercept of the red line with the *x*-axis. Inset: least-squares fitting of the ρ(T) curve of MO10 by Equation (2). The intercept with the *x*-axis gives the value of $T_{BKT}$ (13.06 K, blue arrow).

We have extracted experimentally the $T_{c}$ value in a zero magnetic field, $T_{c0}$, and the sheet resistance in the metallic normal state, $R_{□N}$, just above $T_{c}$, considering a Cooper-pair fluctuation model for a 2D superconducting system developed by Aslamazov and Larkin (AL) [44] and Maki and Thompson (MT) [45,46]. The two parameters were evaluated by a least-squares fitting of the experimental $R_{□}\left(T\right)$ curves (Figure 5), in a *T* range from $T_{c}$ to ≈15 K, by using the relation [13,14]:

(1) $$R_{□}\left(T\right)=\frac{R_{□N}}{1+R_{□N}\frac{\gamma }{16}\frac{e^{2}}{\hbar }\left(\frac{T_{c}}{T-T_{c}}\right)}$$

where: γ is a numerical factor, *ℏ* is the reduced Planck constant, *e* is the electron charge and $T_{c}$ is here intended as the BCS mean-field transition temperature. Fitting was carried out satisfying the condition $ln(T/T_{c})\ll 1$. The result of the $R_{□}\left(T\right)$ fitting of film MO10 is reproduced in Figure 6. Similar findings were found also for MO5a and MO5b.

Fitted and experimental $R_{□N}$ values agree within 2–3%, and $T_{c}$ and γ exhibit a dependence on *d* and the substrate type. This is particularly clear for the $T_{c}$ values for NbN films with 5 nm of thickness (see Figure 5).

In detail, $T_{c}$ values derived by the fit are close, though systematically lower, than those extracted by the analysis of the SC transition branch of the ρ(T) curves (see the Table 1), and the values of γ, ranging from ≈1 to ≈2 (Table 2), are in agreement with those reported for NbN films with d<10 nm [13,14], and in general, comparable with those reported in literature for different SC materials.

Based on theoretical studies, below $T_{BKT}$, all VAPs are bound. While approaching $T_{BKT}$, thermal fluctuations begin to break VAPs, and under a thermodynamic equilibrium, VAPs and single vortices will coexist [10,13,47]. However, due to the sourced current, single vortices will experience a Lorentz force (neglecting vortex pinning), causing the appearance of a finite voltage drop. The resulting film resistivity, in the temperature range $T_{BKT}<T<T_{c0}$, can be described by the relation:

(2) $$\rho \left(T\right)=aexp\left(-2\sqrt{b\frac{T_{c0}-T}{T-T_{BKT}}}\right)$$

where *a* and *b* are fitting parameters related to the SC material, and the values obtained were less than 1 for all films. The $T_{BKT}$ value derived by the least-squares fitting with Equation (2) of the ρ(T) curve of film MO10 is shown in the inset of Figure 6. The fitting procedure was extended to other thin films, for which no such sign of BKT transition was detected. It is interesting to estimate the polarizability, $\epsilon_{BKT}$, of a VAP at the BKT-like vortex phase transition in the presence of other VAPs, by using the relation [13]:

(3) $$\frac{T_{BKT}}{T_{c0}}=\frac{1}{1+0.173\epsilon_{BKT}R_{□N}\frac{2e^{2}}{\pi h}}$$

Equation (3) was applied to NbN films deposited on different substrates, resulting in values of ϵ (Table 2) in close agreement with those found in ref. [13] for 6 nm thick NbN films and also successfully crosschecked with the universal relation $k_{B}T_{BKT}=AT_{BKT}/4\epsilon_{BKT}$ for topological 2D phase transitions; see Nelson and Kosterlitz [48,49]. All the parameters extracted by fitting (see Table 2) are in excellent agreement with the values reported in the ref. [13,14].

**Figure 7:**
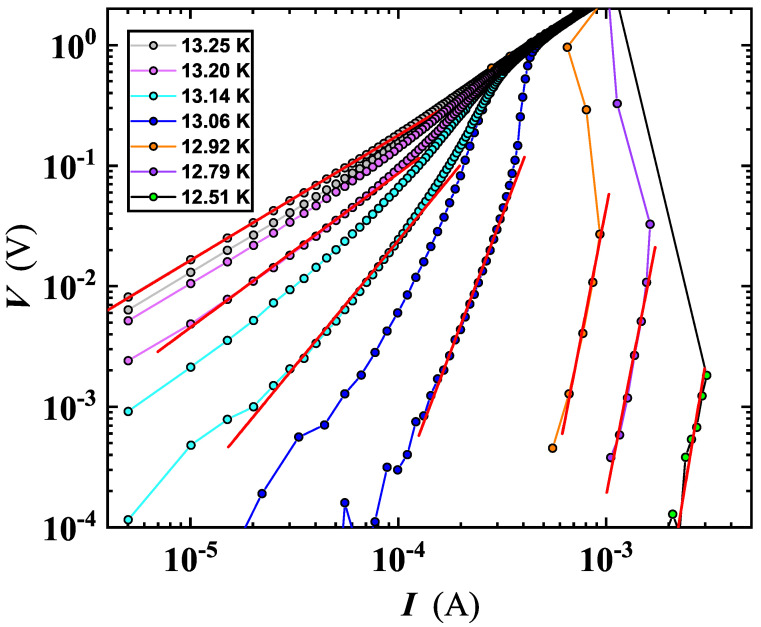
Voltage−current characteristics for the film MO10. Experimental data of the sweep-up curve were fitted by a power law function to extract the value of α (see Equations (4) and (5) and Table 2).

**Figure 8:**
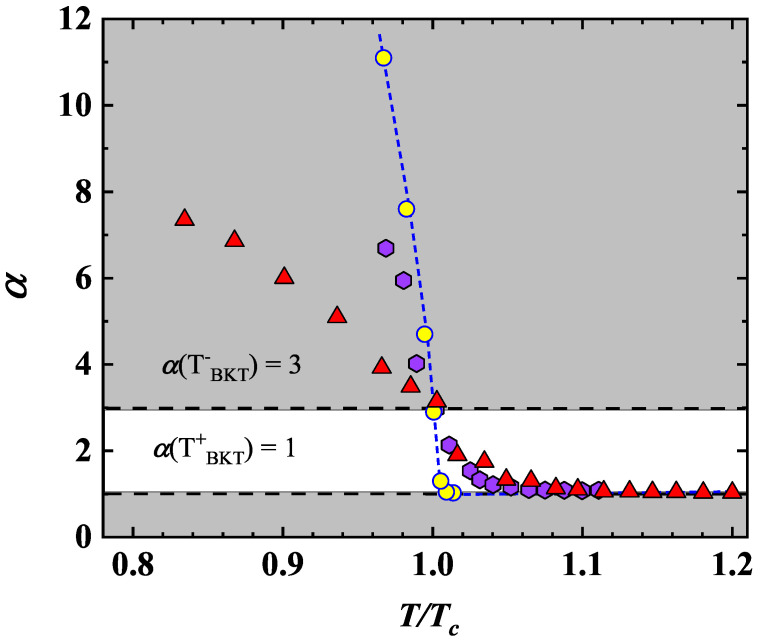
Temperature−dependence of α for MO10 (yellow circle), derived from the power-law fitting of the V-I curves plotted in the Figure 7. A jump in the value of α from ≃1 to ≃3 (white region) was detected at $T_{BKT}$, corresponding to $T/T_{c}=0.989$. For comparison, the α values for a 3 nm thick NbN film from the work of Venditti et al. (purple hexagon) [16] and for Mo$S_{2}$ from the work of Saito et al. (red triangle) [50] were added to the data. The broken line is a guide for the eyes. The temperature was normalized to $T_{BKT}$ for our and Saito et al. data. Venditti et al.’s α values were derived by digitization of Figure (2e) in ref. [16].

To check the possible BKT-like transition, we carried out further analyses considering that thermal fluctuations occurring in ultra-thin films can excite pairs of vortices, each consisting in a single vortex having supercurrents circulating in opposite directions, then leading to the bound vortex anti-vortex pair state [13]. These VAP pairs lead to the specific signature of a BKT-like transition, consisting in a jump in the superconducting stiffness, $J_{s}$, from a finite value, below $T_{c}$, to zero above it. In that case, a non linear dependence exists in the I-V characteristics near $T_{c}$, since large enough currents may unbind VAPs. Hence, due to this effect, a voltage is generated, depending on the equilibrium density of the free vortices, scaling with the sourced current according to a power law, with an exponent proportional to $J_{s}$:

(4) $$V\propto I^{\alpha }\left(T\right)$$

(5) $$\alpha \left(T\right)=1+\pi \frac{J_{s}\left(T\right)}{T}$$

at the BKT transition, the I-V exponent jumps from $\alpha (T_{BKT}^{-}$) = 3 to $\alpha (T_{BKT}^{+})=1$, where the unity value signals the metallic ohmic behavior in the normal state of the I-V characteristics. We have extracted the value of α for the MO10 film, by using Equation (4) to carry out a least-squares fitting of the voltage–current curves, measured at several *T* values (Figure 7). We detected a universal jump from ≃1 to ≃3 at $T_{BKT}$ (Figure 8). Our findings evidence a steeper transition (see Figure 8) resulting in agreement with data of Venditti et al. for a thin film of NbN [16]. The behavior here reported for the NbN system was also found by Saito et al. for a completely different system than the MoS${}_{2}$ [50] (Figure 8), which validates the universal jump in the superfluid density for determining the BKT transition.

### 3.3. Phase Slips

In addition to the BKT transition, interesting outcomes were found in our NbN films in terms of I-V characteristics by the pulse-mode technique (see the Materials and Methods section). In detail, for two of our thinnest films (SR5 and MO5b), we observed the emergence of resistive states as tailing-like features in I-V curves and a double transition in the ρ(T) curves. These findings were interpreted as possible signatures of phase-slip events, arising due to a discontinuous jump by integer multiple of 2π in the phase of the order parameter of the superconducting state, typically existing in quasi-1D systems as nanowires and nanorings. For a discussion of the resistive states associated with PS events in superconducting quasi-1D nanostructures, see ref. [51].

Nevertheless, the investigated NbN films are a 2D system which is granular in nature. The presence of disorder can lead to a weak localization and inhomogeneous effects, resulting in the appearance of a 1D-like features such as PS events [10,11,24].

Taking into account the above-mentioned scenario, such systems are prone to forming an array of continuous conductive paths, having an effective dimensional size much smaller than the physical dimension of the system. In that case, a region equivalent to the coherence length can create a Josephson-like junction, which shows a PS barrier proportional to the area of quasi-1D SC system. Under these circumstances, Cooper pairs will cross the free energy barrier and the relative phase will jump by 2π, resulting in a measurable voltage drop. This drop, will cause a detectable resistance change at $T<T_{c}$, giving rise to PS events driven by thermal and quantum activation [24]. To confirm the possible presence of PS in our films, we took into account the theory of Langer, Ambegaokar, McCumber and Halperin (LAMH), accounting for thermally activated phase slips (TAPS) detected as an effective resistance change related to the time evolution of the superconducting phase:

(6) $$R_{TAPS}=\frac{\pi \hbar^{2}\Omega_{TAPS}}{2e^{2}K_{B}T}exp\left(\frac{-\Delta F}{K_{B}T}\right)$$

where ΔF is the energy barrier to Cooper-pair crossing, and the other quantities have the known meanings. Here, $\Omega_{TAPS}$ is the attempt frequency defined as:

(7) $$\Omega_{TAPS}=\frac{L}{\xi }\left(\frac{\Delta F}{K_{B}T}\right)\frac{1}{\tau_{GL}}$$

where $\tau_{GL}$ is the relaxation time in the time-independent Ginzburg–Landau equation and ΔF is defined as:

(8) $$\Delta F=0.83K_{B}T_{c}\frac{L\beta wdR_{q}}{\rho_{n}\xi_{0}}$$

where β is a fitting parameter, *w* the Hall bar width, *d* the film thickness, $R_{q}=\hbar /2e^{2}≃6.4$ kΩ the quantum of resistance and $\rho_{n}$ the normal state resistivity upon SC transition.

The LAMH theory was originally developed for very long wires, thinner than ξ of the SC material. However, our investigated NbN films had a thickness comparable to ξ. *w* and *L* (i.e., the width and the length of the Hall bar, respectively) were orders of magnitude greater then ξ.

**Figure 9:**
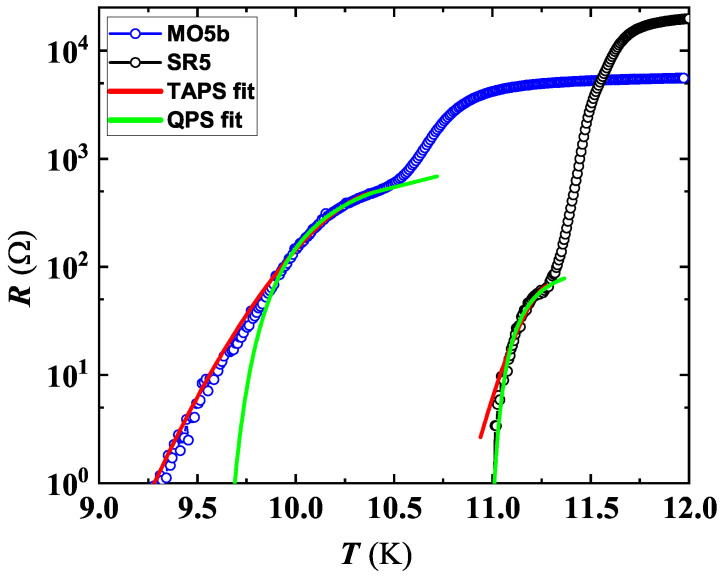
Temperature dependence of resistance for two 5 nm thick NbN films. Both curves showed tailing-like features below about 10.5 and a 11.5 K for MO5b and SR5, respectively. Branches of the ρ(T) curves exhibiting the transition at the lower *T* range were fitted by using LAMH theory, Equation (6) (red lines), and by Equation (9) (green lines).

In order to check for the presence of TAPS in SR5 and MO5b, least-squares fits were carried out by Equation (6), leaving $T_{c}$ and β as fitting parameters. For MO5b, good agreement with the LAMH theory was found, leaving $T_{c}$ and β values comparable to those found in ref. [24] (see Figure 9). Moreover, the $T_{c}$ value obtained by the LAMH fit coincides with that derived from resistance curve analysis (see Table 1), confirming that fluctuation effects are caused by PS and have a thermal origin.

On the other hand, for SR5 at lower *T*, fitting by LAMH theory failed in the first steeper branch of the ρ(T) curve (see Figure 9), suggesting the possibility of a different fluctuation effect present in the same *T* range. To confirm our hypothesis, we explored the possible contribution by QPS emerging from the quantum tunneling of the order parameter through the same free-energy barrier as in TAPS, which is supposed to dominate at lower *T*. The dynamics of the order parameter in the quantum fluctuations were first reported by Giordano [52], suggesting a mechanism similar to TAPS, except that appropriate energy scale $K_{B}T$ is replaced by $\hbar /\tau_{GL}$, resulting in the equation:

(9) $$R_{QPS}=B\frac{\pi \hbar^{2}\Omega_{QPS}}{2e^{2}\frac{\hbar }{\tau_{GL}}}exp\left(-a\frac{\Delta F\tau_{GL}}{\hbar }\right)$$

where *B* and *a* are numerical factors of ≈1 and $\Omega_{QPS}$ is defined as:

(10) $$\Omega_{QPS}=\frac{L}{\xi }\left(\frac{\Delta F}{\frac{\hbar }{\tau_{GL}}}\right)\frac{1}{\tau_{GL}}$$

The result of the fitting by Equation (9) is in agreement with the theoretical predictions for SR5 (see Figure 9), and on the contrary, a progressive deviation from the QPS Equation (9) is evident at lower *T* for MO5b.

The excellent agreement of our experimental results of ρ(T) with the above-mentioned LAMH and QPS models suggests the existence of a nano-conducting path (NCP) having a lateral size comparable to ξ. To estimate this size, we have used the equation from the model developed by Joshi et al. [53]:

(11) $$d_{NCP}=\sqrt{\left(\frac{12\rho_{RT}C\xi_{0}}{1.76\sqrt{2}\pi R_{q}}\right)}$$

where $\xi_{0}≃4$ nm, C=8 (value typically used for quantum systems) [2] and $\rho_{RT}$ is the resistivity value at RT. NCP values calculated by using Equation (11) were 3.2 nm for SR5 and 7.5 nm for MO5b, which are comparable with those found for NbN nanostructures in ref. [53]. In fact, for SR5, the condition $d_{NCP}<\xi$ (i.e., ξ≈4 nm) can be considered as the origin of quantum tunneling, resulting in the formation of QPS detected in our film. On the contrary, for MO5b, $d_{NCP}>\xi$ TAPS lines will result, as indeed experimentally observed.

**Figure 10:**
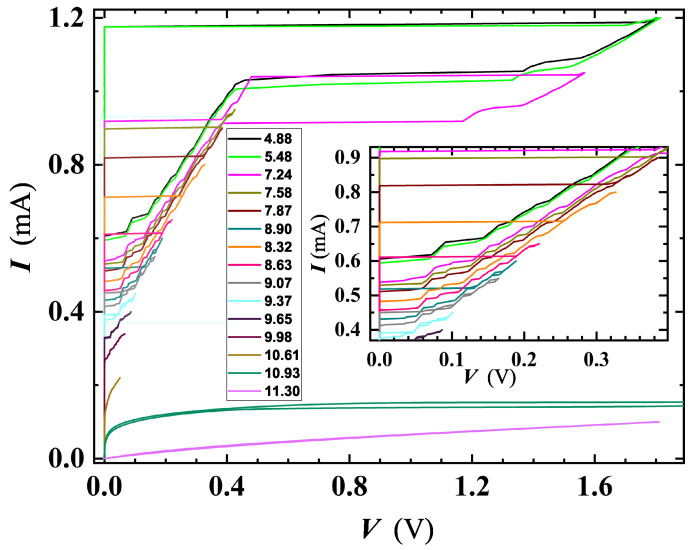
I-V characteristics for the SR5 film, carried out at several *T*. Both up- and down-current sweeps evidence the presence of slanted steps due to the occurrence of intermediate resistive regimes before the complete transition to the normal state. Inset: magnification of the central part of the plot.

**Figure 11:**
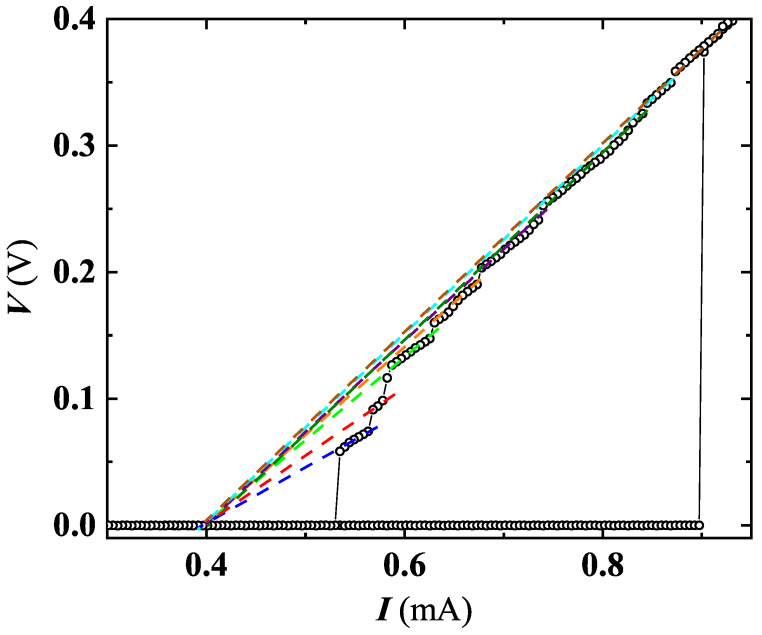
V-I curve at T = 7.48 K is shown for SR5, in which an intercept (represented by dotted lines of different colors for different PSL) on x-axis converge towards a well-defined value of the excess current of $I_{ex}=0.38$ mA.

An interesting outcome derived by a detailed analysis of I-V characteristics carried out on SR5 and MO5b confirmed the existence of PS events. Figure 10 shows a family of I-V curves measured at different *T* for SR5, where multiple slanted steps having resistive tailing-like features were detected. The dynamic resistance of these tails rises at increasing *V* (e.g., along the I-V sweep-up direction, see Figure 11), and the current axis intercept (extending tails slope) occurs for a common current value of $I_{ex}≃$ 0.38 mA, defined as excess current. These findings can be considered as a proof that detected resistive states are originated by PS [54].

**Figure 12:**
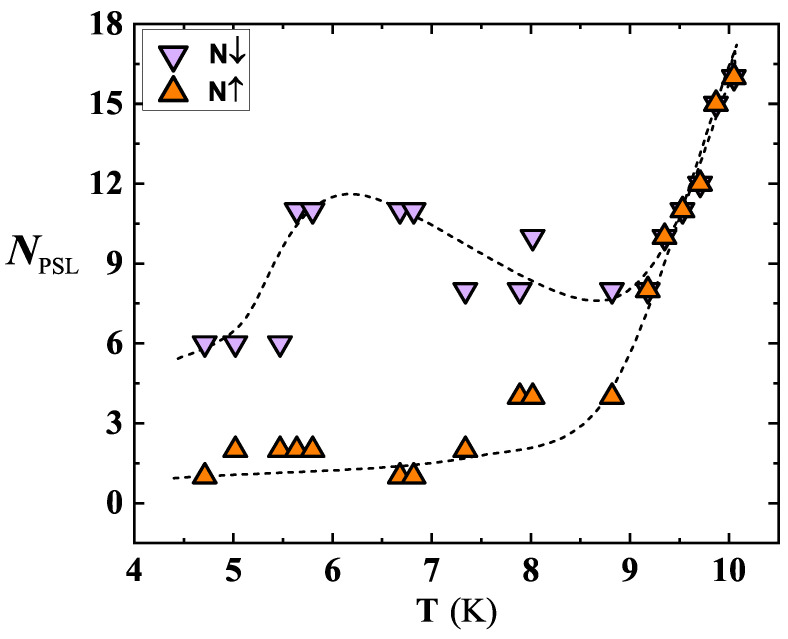
Number of resistive states for SR5, calculated from the I-V curves, during the current sweep-up ($N^{↑}$) and sweep-down ($N^{↓}$). Near to T≃9 K, $N^{↑}$ and $N^{↓}$ suddenly converge, assuming the same value at T≥9 K. Broken lines are guides for the eye.

Further analysis of the I-V curves evidenced interesting temperature dependence of the number of the resistive states appearing in the sweep-up ($N^{↑}$) and sweep-down ($N^{↓}$) branches of the I-V curves (Figure 12). The $N^{↑}$ and $N^{↓}$ kept an almost constant value up to $T\approx T_{c}/2$, and then they started to differ. In detail, at $T≳T_{c}/2$, $N^{↓}$ jumps to higher values, and then it decreases till about 9 K. On the contrary, $N^{↑}$ continues to gradually increase till 9 K. Onward from 9 K, both curves are perfectly overlapped and continuously rise with *T* (Figure 12). The behavior exhibited by $N^{↑}$ and $N^{↓}$ allows one to derive additional observations about fluctuation effects involved in our investigated film. Specifically, the unequal distribution of PS in $N^{↑}$ and $N^{↓}$ suggests that different mechanisms are contributing in three distinct *T* ranges. Below $T_{c}/2$, the emergence of phase-slip events is driven by quantum tunneling. In the intermediate *T* range ($T_{c}/2<T<9$ K), the behavior of $N^{↑}$ and $N^{↓}$ can be explained by the competition of QPS and TAPS, the former tending to decrease approaching 9 K. Finally, above 9 K, only TAPS contributes in the observation of PS events (Figure 12). It is interesting to note that in the region where QPS is present, $N^{↑}$ and $N^{↓}$ diverge. This effect can be explained by a presumably different tunneling route followed by the system, during the sweep up and sweep down of the sourced current in the quantum regime. In the intermediate *T* range, the system self organizes in order to converge towards a condition dominated by TAPS, then leading to a decrease in $N^{↓}$ with an increase $N^{↑}$. Finally, approaching $T_{c}$, thermal fluctuations dominate over QPS, and the entire system is driven by the electrons. Since the electrons tend to track the same path while going sweep-up and sweep-down [55], the number of PS becomes equal, followed by a total increase in the number of PSL, due to enhanced fluctuation effects near $T_{c}$.

**Figure 13:**
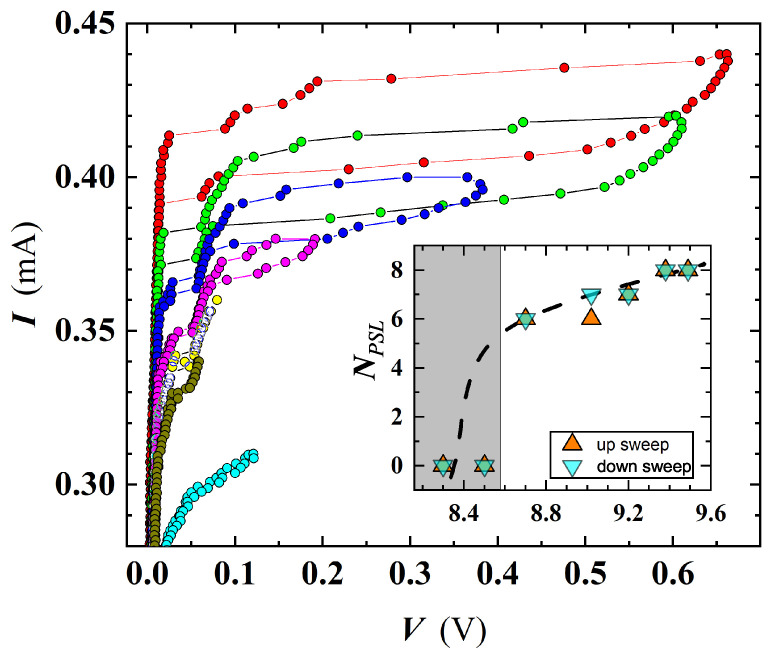
I-V characteristics of MO5b, showing the occurrence of steps in the curves close to $T_{c}$, progressively disappearing approaching $T_{c}$. The *T* values for the shown curves are: 8.70 K (red), 9.02 (green), 9.20 K (blue), 9.38 K (magenta), 9.49 K (yellow), 9.60 K (olive), 9.98 K (cyan). Inset: number of TAPS extracted from I-V curves. The gray rectangle defines the *T* range where no PSL were detected.

In addition to TAPS, BKT-like features were also observed in MO5b (see Figure 13), in the *T* range where PS disappears and the system still remains in the SC state. Moreover, in the same film, BKT was confirmed by the scaling behavior of Halperin–Nelson equation above $T_{BKT}$ (as explained in the BKT section). Interestingly, with our I-V characteristics, the TAPS completely disappears at 9.9 K, far before reaching $T_{c}$ (11.02 K, see Table 2), and this PS suppression gives rise to BKT-like transition, i.e., an effect typical of a 2D system, which involves breaking of VAP. For $T>T_{BKT}$, the unbinding of VAP will result in the universal jump in α, as already detected in the I-V characteristics of MO10 (see Figure 7). The parameters derived for this film (see Table 2) are in good agreement with the theory. We believe that the inhomogeneity of the NbN system contributed to the origins of these experimental findings. However, our results suggest that the level of inhomogeneity in the studied system is just sufficient to create a thermal fluctuation which results in the emergence of TAPS but not too strong to destroy the superconductivity of the film. The detection of BKT in the same film seems to confirm this hypothesis, since higher inhomogeneity would have destroyed the BKT effect. The coexistence of BKT and TAPS in the same system, the former being typical in a 2D system and the latter in a quasi-1D system, suggests that the internal structure of our investigated films is on the boundary line of a 2D–1D dimensional crossover.

**Figure 14:**
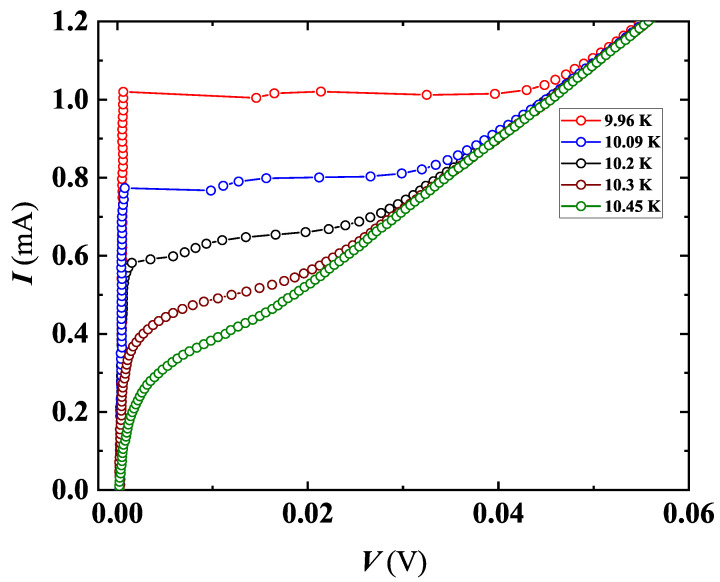
I-V characteristics for SO5, at selected *T* values close to $T_{c}$. Less-marked resistive tails are now visible, whose values are reduced if compared to those detected in the film SR5 (see the Figure 10).

Similar findings were measured at 5 nm of thickness for NbN films deposited on other types of substrates. However, on SiO${}_{2}$, resistive tails appeared less pronounced due to the larger width of the Hall bar (see Figure 14).

## 4. Conclusions

In summary, our study of the superconducting properties of NbN nanofilms showed several features associated with complex phase fluctuations of the order parameter. Resistivity and I-V curves showed a well-defined BKT transition to the superconducting state characteristic of 2D systems. In addition to the BKT physics, we also detected and characterized phase-slip events (both quantum and thermal) typical of quasi-1D superconductors. Both effects deman careful fine tuning of the experimental set-up and material system.

To analyze the BKT transition, we used the Cooper pair fluctuation model. We found that the linear in *T* dependence of the resistivity above $T_{c}$ is only compatible with 2D fluctuation-conductivity and incompatible with the predictions for 1D and 3D systems, confirming the 2D dimensionality of our NbN films. Our findings of polarizability values of VAP at the BKT transition are in good agreement with the Nelson and Kosterlitz universal relation in two different NbN films. In one case, the polarizability was found to be almost twice the expected value. This evidences the 3D to 2D dimensional crossover at 10 nm, since no BKT transition was detected at 15 nm. Further confirmation is given by the α exponent extracted from I-V curves, exhibiting a steep transition from 1 to 3, close to $T_{BKT}$, in agreement with theory.

Regarding the PS events, in one sample we detected both quantum and thermal PSs, in different *T* regimes. QPSs depend on the quantum tunneling route undertaken by the system. These outcomes were explained by the presence of granularity in NbN. A careful analysis of film resistivity suggested the presence of a nano-conductive path, making NbN films equivalent to a quasi-1D system, explaining the presence of PS events. We investigated specific features of PS events as the numbers of PS occurring during the up-sweep and down-sweep sourced current. The distribution of PSs in the quantum regime is uneven, though converging on the same value in the *T* regime dominated by thermal fluctuations. Moreover, we have evidenced the co-existence of BKT and PS phenomena in the same NbN nanofilm, which has not been reported until now. Considering that BKT and PS events belong to two different dimensionality systems, this means that we have successfully addressed a 2D to quasi-1D dimensional crossover in the same system. Finally, our experimental findings motivate us for in depth study of superconducting NbN nanofilms as a tunable platform to generate and control novel quantum phenomena exploitable for quantum technologies. 

## Figures and Tables

**Table 1:** NbN film’s properties. Starting from the left, columns are: film acronym (SC: Al${}_{2}$O${}_{3}$ c-cut; SR: Al${}_{2}$O${}_{3}$ r-cut; MO: MgO; SO: SiO${}_{2}$; the number following the two letters refers to the film thickness in nm units); resistivity value at 15 K; superconducting transition temperature; superconducting transition width; superconducting critical-current density at 0 K.

Sample	ρ (Ω cm)	$T_{c}$ (K)	$\Delta T_{c}$ (K)	$J_{c0}$ (MA/cm${}^{2}$)
MO5a *	8.0 × $10^{-5}$	10.072	0.08	0.40 ± 0.014
MO5b **	5.8 × $10^{-4}$	11.02	0.79	2.24 ± 0.013
MO10	1.2 × $10^{-4}$	13.29	0.27	9.98 ± 0.15
MO15	2.4 × $10^{-4}$	13.83	0.23	11.40 ± 0.016
SC5	8.0 × $10^{-5}$	10.64	0.43	0.90 ± 0.010
SC10	2.4 × $10^{-4}$	13.50	0.40	10 ± 0.028
SC15	1.7 × $10^{-4}$	12.73	0.24	5.29 ± 0.08
SR5 ^**^	1.1 × $10^{-4}$	11.76	0.68	0.63 ± 0.013
SR10	1.7 × $10^{-4}$	12.43	0.30	8.3 ± 0.18
SR15	2.3 × $10^{-4}$	12.58	0.38	6.14 ± 0.13
SO5	9.3 × $10^{-7}$	9.40	0.46	0.89 ± 0.024

* Both MO5a and MO5b belonged to the same deposition run, but the fabrication process of their Hall bar was carried out by using slightly different parameters. ** The Hall bar width of this film was 10 µm.

**Table 2:** Berezinskii–Kosterlitz–Thouless (BKT) parameters derived by the analysis of the resistivity and I-V characteristics curves of some of the thinner NbN films. Column headings, from left: sample acronym *; BKT temperature derived by ρ(T) fitting with Equation (2); SC transition temperature at *B* = 0 (see the text); normal state sheet resistance at 20 K; γ value (see the text and Equation (1)); VAP polarizability.

Sample	$T_{\mathrm{BKT}}$ (K)	$T_{c0}$ (K) **	$R_{□N}$ (Ω)	γ	ϵ
MO5a	9.70 ± 0.03	9.90	502.30 ± 0.01	0.970 ± 0.002	10.28 ± 0.030
MO5b	10.30 ± 0.02	10.60	620 ± 1.06	1.500 ± 0.005	11.49 ± 0.022
MO10	13.060 ± 0.008	13.31	129.8 ± 0.60	1.56 ± 0.09	25.5 ± 0.11

* For meaning of sample acronym see the caption of Table 1. ** The fitting error on $T_{c0}$ is of the order of 10^−4^.

## Data Availability

Not applicable.

## Abbreviations

The following abbreviations are used in this manuscript:

SC	Superconducting
BKT	Berezinskii–Kosterlitz–Thouless
PS	Phase slip
TAPS	Thermally activated phase slips
QPS	Quantum phase slips
NCP	Nano-conducting path
